# Integrated Phylogenomics and Expression Profiling of the Peptide Deformylase Gene Family in *Oryza sativa* Reveals Their Role in Development and Stress Tolerance

**DOI:** 10.3390/cimb48040396

**Published:** 2026-04-13

**Authors:** Chen Yuan, Yanli Zhang, Minghui Zhao, Dianrong Ma

**Affiliations:** 1 Rice Research Institute, Shenyang Agricultural University, Shenyang 110161, China; rachel94yc@163.com (C.Y.); mhzhao@syau.edu.cn (M.Z.); 2 College of Life and Environmental Science, Hangzhou Normal University, Hangzhou 311121, China; zhangyanli1121@sina.com; 3 Liaoning Academy of Agricultural Sciences, Shenyang 110161, China

**Keywords:** peptide deformylase, bioinformatics analysis, *Oryza sativa*, abiotic stress

## Abstract

Peptide deformylase (PDF) belongs to a conserved enzyme family critical for N-terminal methionine excision (NME), an essential protein maturation process in prokaryotes and eukaryotic organelles (chloroplasts, mitochondria). To explore the potential functions of *OsPDFs* in *Oryza sativa*, this study employed bioinformatics approaches and experimental validation to systematically identify and analyze the *OsPDF* gene family. Three *OsPDF* genes (*OsPDF1A*, *OsPDF1B*, *OsPDF1B2*) were identified in rice. These genes are exclusively distributed on chromosome 1. The biophysical properties of these proteins showed that OsPDF1A and OsPDF1B are alkaline proteins, while OsPDF1B2 is acidic, and all are hydrophilic with moderate thermostability potential. Synteny analysis revealed closer evolutionary relationships between *Oryza sativa* and the monocot *Triticum aestivum* than with dicots, reflecting conserved PDF function in gramineous plants. Analysis of cis-acting elements in the 2000 bp upstream region of *OsPDF* gene promoters revealed numerous elements associated with abiotic stress response and hormone regulation. Furthermore, quantitative real-time PCR (qRT-PCR) data supported these findings, indicating that *OsPDF1A* and *OsPDF1B* were upregulated under low-temperature stress, and all three *OsPDF* genes were transcriptionally activated by heat, salt and UV-B stresses, indicating their active involvement in rice growth, development, and abiotic stress tolerance. In summary, OsPDFs exhibit significant functions in rice’s stress adaptation, growth, and development. This study not only enhances our understanding of the *OsPDF* gene family’s genomic, evolutionary, and functional characteristics, but also provides new perspectives and foundational data for further exploring their regulatory mechanisms in protein maturation and abiotic stress responses, as well as their potential applications in rice stress tolerance breeding.

## 1. Introduction

Peptide deformylase (PDF) is a conserved enzyme family that plays a critical role in N-terminal methionine excision (NME), which is a fundamental protein maturation process conserved across prokaryotes and the organelles (chloroplasts and mitochondria) of eukaryotes. Its core function is to catalyze the removal of the formyl group from the N-terminal formylmethionine (fnMet) of nascent polypeptides. This step is a prerequisite for the subsequent cleavage of methionine by methionine aminopeptidase (MAP), which in turn ensures the proper localization, stability, and activity of proteins [1,2,3].

In bacteria, protein synthesis initiates with formylated methionine-tRNA (fMet-tRNA_f_^Met^), where the formyl group is added by methionyl-tRNA formyltransferase (FMT) using 10-formyltetrahydrofolate (10-fTHF), which is a metabolite synthesized via FolA (dihydrofolate reductase) and FolD (methylenetetrahydrofolate dehydrogenase) [4,5]. PDF then removes this formyl group from approximately 95% of bacterial proteins (excluding secreted proteins with signal peptides), a step that enables subsequent NME mediated by MAP [6]. PDF is a well-validated antimicrobial target, and this role is linked to the biological activity of formylated peptides. These byproducts of bacterial protein synthesis act as pathogen-associated molecular patterns (PAMPs), binding to formyl peptide receptors (FPRs) on mammalian leukocytes to trigger proinflammatory responses, such as chemotaxis and reactive oxygen species production [7,8]. Inhibitors like actinonin (a potent PDF antagonist with nanomolar affinity) disrupt the NME process, leading to the accumulation of misfolded proteins, membrane defects, and ultimately bacterial cell death [9,10]. However, bacterial resistance to PDF inhibitors has emerged, primarily through mutations in the *fmt* gene (which encodes FMT), which bypasses the need for formylation, thereby rendering PDF inhibition functionally irrelevant [11]. Notably, genome-reduced pathogens (e.g., mycoplasmas) exhibit unique adaptive traits related to this pathway. A bioinformatic analysis of 54 mycoplasma species (including the swine pathogen *Mycoplasma hyopneumoniae*) revealed 14 species (all *haemoplasmas* and a *Hominis* clade) lack FolA, FolD, FMT, and PDF [12]. LC-MS/MS analysis further confirmed the absence of N-terminal formylmethionine (fnMet) in *M. hyopneumoniae* proteins, a stark contrast to *Mycoplasma pneumoniae* (a human pathogen with intact NME machinery). Additionally, the translation initiation factors (IF1, IF2, IF3) of *M. hyopneumoniae* display structural irregularities. For instance, IF2 lacks critical residues involved in fMet-tRNA_f_^Met^ binding, which reduces their affinity for fMet-tRNA_f_^Met^. These features collectively support an evolutionary shift in *M. hyopneumoniae* toward using unformylated methionine (nMet) for translation initiation, an adaptation likely driven by the need to evade host immune detection [12].

In eukaryotes, cytoplasmic protein translation initiates with unformylated methionine. However, chloroplasts and mitochondria (endosymbiotic descendants of prokaryotes) retain the prokaryotic trait of fnMet-initiated protein synthesis, which requires organelle-targeted PDFs to complete protein maturation [13]. Plant PDFs of type 1 (PDF1) are classified into two subgroups based on subcellular localization: PDF1A, which targets mitochondria, and PDF1B, which localizes to chloroplasts, mitochondria, or both. For instance, *Oryza sativa* OsPDF1B is dual-localized to both organelles [14], whereas *Eucommia ulmoides* EuPDF1B (the first characterized PDF1B from a woody plant) is exclusively chloroplastic. The localization pattern of EuPDF1B is proposed to be a woody-plant-specific adaptive trait [15]. PDF1B plays a critical role in chloroplast development and function. In rice, *pdf1b* mutants exhibit visible chlorosis (yellowish leaves), stunted growth, and severe chloroplast damage-including disorganized thylakoid membranes, which are essential for photosynthesis [14]. In tobacco, inhibiting PDF activity with actinonin (a potent PDF inhibitor) reduces the accumulation of the D1 protein, a core component of photosystem II (PSII) encoded by the chloroplast *psbA* gene, ultimately leading to PSII degradation and leaf death [16,17]. For *E. ulmoides* EuPDF1B, expression analysis reveals tissue-specific and environment-responsive patterns. Its transcript levels are highest in mature leaves (consistent with a role in photosynthesis) and are induced by the hormones abscisic acid (ABA), methyl jasmonate (MeJA), and gibberellin (GA), but suppressed by shading. These expression patterns correlate with cis-acting elements identified in the *EuPDF1B* promoter, such as ABA-responsive elements (ABREs) and MeJA-responsive CGTCA motifs [15]. Functional validation in transgenic tobacco further confirms EuPDF1B’s role in PSII maintenance. Plants overexpressing *EuPDF1B* exhibit increased *psbA* gene expression, enhanced net photosynthetic rate, and increased plant growth compared to wild-type plants [15].

Plant PDFs exhibit distinct biochemical properties that set them apart from their bacterial homologs, with functional traits tailored to plant organelle-specific protein maturation needs. For instance, recombinant PDF1 proteins from *Arabidopsis thaliana*, pAtDEF1 (PDF1A) and pAtDEF2 (PDF1B) display notable differences in kinetic parameters. pAtDEF2, which targets chloroplasts (and sometimes mitochondria), shows a 240-fold higher catalytic efficiency for the N-terminus of the D1 protein compared to other peptide substrates. In contrast, pAtDEF1 (a mitochondria-targeted PDF1A) has a 2-fold lower binding affinity for actinonin relative to pAtDEF2, reflecting functional specialization linked to their subcellular localizations [17,18]. In woody plants, genome-wide screening of *Populus trichocarpa* (poplar) identified two *PDF1* genes: *PtrPDF1A* and *PtrPDF1B*. The initial sequence of *PtrPDF1B* was truncated, and its full-length sequence was revised using expressed sequence tags (ESTs) to ensure accuracy. Subsequent in silico structural modeling confirmed that PtrPDF1B shares key catalytic features with Arabidopsis PDF1B, indicating that despite evolutionary divergence between herbaceous and woody plants, the core catalytic function of PDF1B is conserved, which further supports the essential role of PDF1B in plant photosynthetic and organelle function [19].

Plant PDFs hold substantial biotechnological value, with applications spanning transgenic selection and herbicide development. Transgenic tobacco studies have demonstrated that overexpression of *Arabidopsis thaliana AtDEF1.2* or *AtDEF2* confers resistance to actinonin. In contrast, overexpression of *AtDEF1.1* (a mitochondria-targeted PDF1A) fails to induce such resistance, confirming that chloroplast localization of PDFs is critical for mediating actinonin tolerance [20]. Notably, actinonin resistance in these transgenic lines cosegregates with kanamycin resistance, a marker typically used for transgenic screening [20]. This finding validates PDFs as effective native selectable markers for plant transformation, which avoids the potential risks associated with transferring antibiotic resistance genes into the environment or food crops. Beyond transgenic selection, PDFs also serve as promising targets for herbicide development. Actinonin inhibits growth across diverse plant species, including agricultural weeds, by disrupting chloroplast function, which is driven by its inhibition of chloroplast-localized PDFs [20]. Importantly, structural differences between plant and bacterial PDFs (e.g., Arabidopsis AtDEF2 is uniquely conserved in plant plastids and *Apicomplexa*, with no direct bacterial homologs) enable the design of plant-specific PDF inhibitors. Such inhibitors can target weed PDFs without exerting antimicrobial activity against beneficial or pathogenic bacteria, reducing unintended ecological impacts and ensuring biosafety [20,21].

In this study, we comprehensively analyzed three *OsPDF* members in *Oryza sativa*. We characterized their structural features and chromosomal distribution and constructed a multi-species phylogenetic tree, and the results revealed that the *OsPDF* gene family has evolved into two distinctly divergent subfamilies, PDF1A and PDF1B, over long-term evolution. In addition, synteny analysis revealed closer evolutionary relationships between *Oryza sativa* and *Triticum aestivum*. qRT-PCR analyses exhibited stress-specific expression patterns among the *OsPDF* genes, with distinct regulatory profiles suggesting functional specialization in response to multiple abiotic stresses. Our findings not only fill a critical knowledge gap in the research on the *OsPDF* gene family but also establish a solid foundation for investigating the potential roles of *OsPDFs* in mediating stress tolerance mechanisms in *Oryza sativa*.

## 2. Materials and Methods

### 2.1. Sequence Alignment and Phylogenetic Analysis of OsPDFs

We used MEGA 11.0.13 software to perform a phylogenetic analysis of the *OsPDF* families of *Oryza sativa*, *Glycine max*, *Zea mays*, *Solanum lycopersicum*, *Triticum aestivum*, *Arabidopsis thaliana*, *Eucommia ulmoides* and *Escherichia coli*. The built-in ClustalW program was used for multiple sequence alignments, and the neighbor-joining (NJ) method with a bootstrap value of 1000 repetitions was used to construct the phylogenetic tree. The iTOL website (https://itol.embl.de/ (accessed on 14 October 2025)) was used to visualize the phylogenetic tree.

### 2.2. Chromosome Location

We drew a chromosome map of *OsPDF* family genes with TBtools v2.363 software. The genome annotation file *Oryza_sativa*.IRGSP-1.0.60.chr.GFF3 used for this was obtained from the website of Ensembl Plants (https://plants.ensembl.org/index.html (accessed on 29 October 2025)).

### 2.3. Synteny Analysis of OsPDF Genes

To investigate the collinearity of *OsPDF* genes with other plant species, we employed the One Step McScanX program in TBtools v2.363 to generate collinear files. Genome data of *Arabidopsis thaliana* (TAIR 10.1), tomato (*Solanum lycopersicum*), soybean (*Glycine max* v2.1), maize (*Zea mays* L. 5.0) and wheat (*Triticum aestivum*) were obtained from https://plants.ensembl.org/index.html (accessed on 29 October 2025). The results were visualized and built by TBtools v2.363.

### 2.4. The Biophysical Properties of OsPDFs

The relative molecular weight (Mw), amino acid (AA) number, and theoretical pI of proteins and the members of the *OsPDF* family gene were calculated by ExPasy (https://www.expasy.org (accessed on 8 November 2025)).

### 2.5. The Identification of Cis-Regulatory Elements in Promoter Regions

To identify the putative cis-acting elements found in *OsPDF* genes, the 2.0 kb genomic sequence upstream of the initiation codon (ATG) of each gene was identified in PlantCARE (https://bioinformatics.psb.ugent.be/webtools/plantcare/html/ (accessed on 16 October 2025)). The results were visualized and constructed using TBtools v2.363.

### 2.6. RNA Isolation and Quantitative Real-Time PCR (qRT-PCR) Analysis

The wild-type seeds were soaked in an incubator at 30 °C, and after 3–4 days, the germinated seeds were sown in a growth chamber. The growth conditions were as follows: temperature 30/22 °C (day/night), relative humidity 60–70%, light cycle 12 h/12 h (day/night), and light intensity approximately 200 µmol m^−2^s^−1^.

For gene expression analysis, 10-day-old wild-type seedlings were exposed to simulated temperature stress with the growth condition at 10 °C or 45 °C, salt stress with a 160 mM NaCl solution, and UV-B stress with UV-B treatment.

Samples were collected after each treatment for subsequent RNA extraction. Total RNA was extracted using an RNAprep Pure Plant Total RNA extraction kit (TIANGEN, Beijing, China). After cDNA Synthesis with the FastKing gDNA Dispelling RT SuperMix (TIANGEN, Beijing, China), qRT-PCR was performed using the UltraSYBR Mixture (Cwbio, Taizhou, China) with a CFX Opus 96 real-time PCR system (BIO-RAD, Hercules, CA, USA). The reaction system was as follows: 0.25 µL of each forward and reverse primer, 1 µL of cDNA, 5 µL of 2× UltraSYBR Mixture, and 3.5 µL of ddH_2_O. The amplification program was as follows: pre-denaturation at 95 °C for 5 min; followed by 40 cycles of denaturation at 95 °C for 10 s and annealing/extension at 60 °C for 25 s. The melting curve program was as follows: 95 °C for 15 s, 60 °C for 1 min, and 95 °C for 15 s. Rice Actin was used as the internal reference gene, and the relative expression level was calculated and analyzed using the 2^−∆∆Ct^ approach. Three biological replicates and three technical replicates were set up for gene expression analysis. The primers used in the experiment are listed in Table S1.

## 3. Results

### 3.1. Peptide Deformylase Genes in Rice

**Figure 1:**
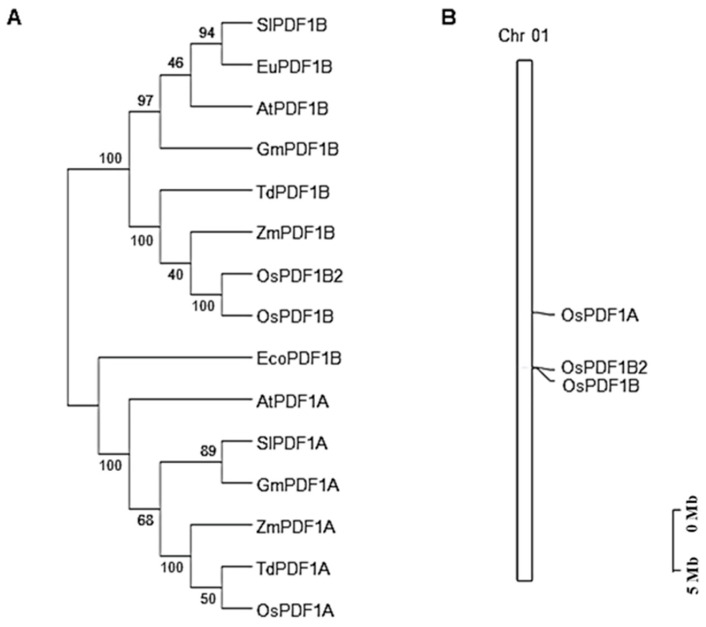
(**A**) Phylogenetic analysis of the PDF family genes of *Oryza sativa*, *Glycine max*, *Zea mays*, *Solanum lycopersicum*, *Triticum aestivum*, *Arabidopsis thaliana*, *Eucommia ulmoides* and *Escherichia coli*. The protein sequence alignments and construction of the phylogenetic tree were performed using MEGA 11.0.13 and the neighbor-joining method with 1000 bootstrap replicates. Branches indicate different evolutionary clades. (**B**) Chromosome locations of *OsPDF* family genes. The length of the bars indicates the sizes of *Oryza sativa* chromosomes. The genes are labeled on the right of the chromosomes.

A previous study identified three peptide deformylase genes in *Oryza sativa* [14]. To further clarify the evolutionary relationships among these *PDF* family members, we performed comparative sequence alignments of *OsPDF* genes with their orthologs from other species. Subsequently, an unrooted phylogenetic tree was constructed using MEGA 11.0.13 software. As presented in Figure 1A, the PDF family is clustered into two distinct subfamilies: OsPDF1A is classified into the PDF1A subgroup, while OsPDF1B and OsPDF1B2 are grouped into the PDF1B subgroup. To intuitively visualize the chromosomal localization of *OsPDF* genes in *Oryza sativa*, we generated a chromosomal distribution map using TBtools v2.363. Notably, all three *OsPDF* genes were found to be localized on chromosome 1 (Figure 1B).

### 3.2. Gene Motif and Structure of the OsPDFs

**Figure 2:**
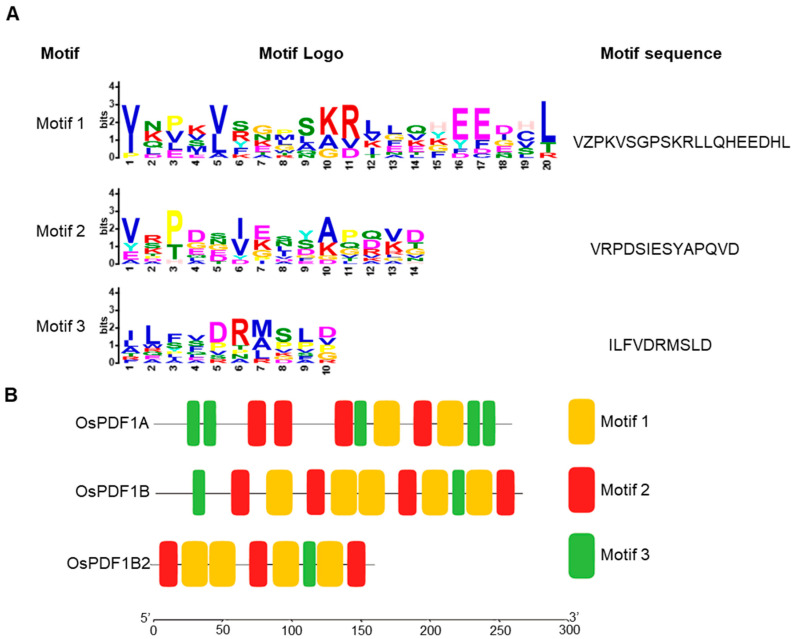
(**A**) Sequences of three motifs identified by MEME v5.5.9. The MEME v5.5.9 tool was used with the following parameters: protein; nostatus; mod anr; nmotifs 6; minsites 17; minw 10; maxw 100. (**B**) Sequence logo representation of motifs defined from the OsPDF proteins by MEME.

In *Oryza sativa*, PDF proteins harbor three highly conserved motifs, which are critical for their catalytic function (Figure 2A). A visual analysis of motif distribution across the three OsPDF proteins (OsPDF1A, OsPDF1B, and OsPDF1B2) revealed distinct patterns in the number of Motif3 copies (Figure 2B). Specifically, OsPDF1B and OsPDF1B2 contain only one or two instances of Motif3 along their amino acid sequences, whereas OsPDF1A possesses approximately five copies of Motif3, which account for half of all motifs in its protein sequence (Figure 2B). These differences in motif distribution may underlie the functional divergence and subcellular localization variations observed among the three OsPDF proteins.

### 3.3. Synteny Analysis of OsPDFs Genes

**Figure 3:**
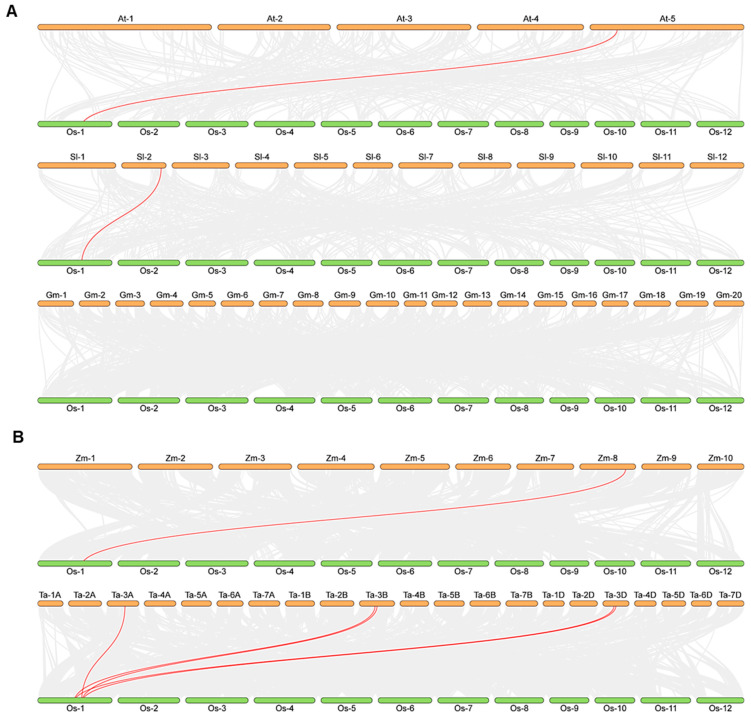
Analysis of syntenic relationships between *Oryza sativa* and different species. (**A**) *Arabidopsis thaliana*, *Solanum lycopersicum* and *Glycine max*; (**B**) *Zea mays* and *Triticum aestivum*. Gray line represents the syntenic block in plant genomes, and red line represents the collinear *PDF* gene pair.

To further explore the evolutionary relationships within the *OsPDF* family, we constructed a gene collinearity map for *Oryza sativa* and compared its *OsPDF* genes with those of five representative species, categorized into dicotyledons (*Arabidopsis thaliana*, *Solanum lycopersicum* and *Glycine max*) and monocotyledons (*Zea mays* and *Triticum aestivum*). The synteny analysis results showed distinct patterns of collinear *PDF* gene pairs between *Oryza sativa* and the five species: only one collinear pair was identified between *Oryza sativa* and either *Arabidopsis thaliana* or *Solanum lycopersicum*, while no collinear pairs were detected between *Oryza sativa* and *Glycine max* (Figure 3A). Among monocot comparisons, *Oryza sativa* shared one collinear pair with *Zea mays* and five collinear pairs with *Triticum aestivum* (Figure 3B). Notably, the number of orthologous *PDF* genes between monocot species was significantly higher than that between monocots and dicots—an observation consistent with established biological evolutionary patterns. Furthermore, the highest number of collinear *PDF* gene pairs was found between *Oryza sativa* and *Triticum aestivum*, suggesting that these two monocot species may share a closer evolutionary history.

### 3.4. The Biophysical Properties of OsPDF Proteins

We analyzed the key biophysical properties of three OsPDF proteins in *Oryza sativa*, with the results summarized in Table 1. In terms of molecular weight, distinct differences were observed among the three proteins: OsPDF1A has a molecular weight of 27.493 kDa, OsPDF1B is slightly larger at 29.676 kDa, and OsPDF1B2 is significantly smaller, weighing 18.007 kDa. Their isoelectric points (pI) also vary notably: OsPDF1A and OsPDF1B have pI values of 8.79 and 8.54, respectively, while OsPDF1B2 has a pI of 5.47. Based on these pI values, OsPDF1A and OsPDF1B are classified as alkaline proteins, while OsPDF1B2 is acidic. Stability predictions indicate that all three OsPDF proteins are unstable, as their instability indices exceed 45. For the aliphatic index (an indicator of potential thermostability), OsPDF1B2 exhibits the highest value (97.89), followed by OsPDF1A (96.81) and OsPDF1B (93.53), suggesting that the family has moderate to strong thermal stability potential. Additionally, the Grand Average of Hydropathicity index for each OsPDF protein is less than 0, which reflects their inherent hydrophilic nature.

### 3.5. Cis-Regulatory Element Identification in OsPDF Promoters

**Figure 4:**
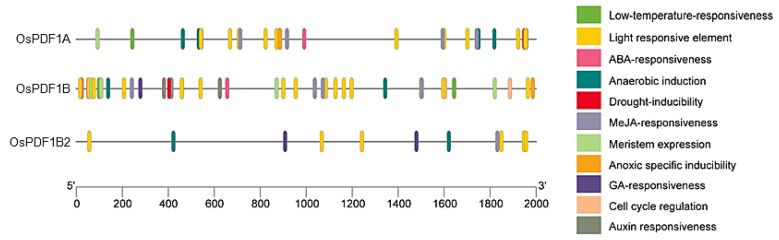
Cis-element analysis in the promoter sequences of *OsPDF* genes. Visualization of the cis components of the *OsPDF* family with TBtools.

Cis-acting elements in promoter regions are pivotal for regulating the expression of their corresponding genes, as they mediate interactions with various transcription factors. To investigate the regulatory potential of *OsPDF* genes, we analyzed the 2000 bp sequences upstream of their start codons using the PlantCARE database to identify cis-regulatory elements, and visualized the results via TBtools v2.363 (Figure 4).

All three members of the *OsPDF* gene family contain cis-acting elements associated with responsiveness to ABA, MeJA, GA, and auxin. Specifically, *OsPDF1B* harbors all cis-elements corresponding to these four hormones, while *OsPDF1A* contains only those linked to ABA and MeJA, and *OsPDF1B2* carries elements associated with MeJA and GA. This differential distribution of hormone-responsive elements suggests that *OsPDF* genes may participate in a diverse range of biological processes modulated by these phytohormones. In addition to hormone-responsive elements, the promoters of *OsPDF* genes also contain light-responsive cis-elements, metabolism-regulatory elements, and cell cycle-regulatory elements. These findings imply potential roles of *OsPDFs* in regulating plant growth and development, as well as mediating light-dependent physiological pathways. Furthermore, multiple stress-related cis-elements were identified, including those involved in low-temperature-responsiveness, anoxic inducibility, anaerobic induction, and drought-responsiveness. These elements collectively indicate that *OsPDF* genes might play important roles in enhancing rice’s adaptability to adverse environmental conditions. Overall, this promoter analysis reveals that the *OsPDF* gene family has evolved a modular combination strategy of cis-acting elements, enabling multi-dimensional responses to hormonal signals, growth cues, and environmental stresses. Among the identified cis-elements, the functional roles of *OsPDFs* linked to core regulatory elements (e.g., low-temperature-responsive elements) may be prioritized for further experimental validation, thereby helping to elucidate their specific contributions to rice physiology and stress adaptation.

### 3.6. Transcriptional Response of OsPDF Genes to Abiotic Stress

Abiotic stresses exert profound impacts on plant growth and development. To elucidate the regulatory effects of temperature (low and high), salt stress, and UV-B radiation on the expression of *OsPDF* genes, we systematically analyzed their transcriptional responses under these stress conditions using quantitative real-time PCR (qRT-PCR).

**Figure 5:**
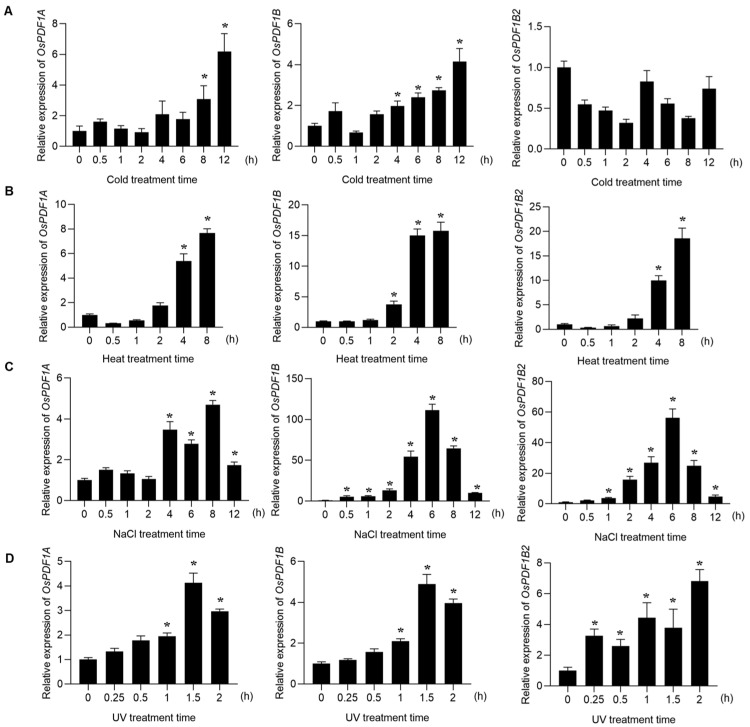
The relative expression analysis of the *OsPDFs* under abiotic stresses. (**A**) Low-temperature treatment, (**B**) high-temperature treatment, (**C**) salt treatment, (**D**) UV-B treatment (n  =  3, mean  ±  SD) (Student’s *t* test, *, *p* < 0.001).

Following low-temperature treatment, *OsPDF1A* and *OsPDF1B* exhibited significant upregulation, whereas *OsPDF1B2* maintained a nearly stable expression level (Figure 5A). In contrast, all *OsPDF* genes were transcriptionally activated under heat stress, with their expression levels peaking at 8 h post-treatment (Figure 5B). Under salt stress, all *OsPDF* genes showed increased expression after treatment. Notably, *OsPDF1B* displayed more dramatic upregulation, with significant upregulation detected at 6 h post-salt treatment (Figure 5C). Additionally, UV-B irradiation induced the upregulation of all three *OsPDF* genes, with their expression reaching maximum levels at either 1.5 h or 2 h after treatment (Figure 5D). Collectively, these results demonstrate that members of the *OsPDF* gene family are universally involved in these stress responses. However, the underlying molecular mechanisms governing their regulatory roles in these stress response pathways require further investigation.

## 4. Discussion

Peptide deformylase is a key enzyme in the N-terminal methionine excision pathway, which is critical for protein maturation across prokaryotes and eukaryotic organelles (such as chloroplasts and mitochondria) [1,2,13]. In rice, three *PDF* genes were identified, *OsPDF1A*, *OsPDF1B*, and *OsPDF1B2*. Prior studies have shown that these genes exhibit distinct expression patterns and subcellular localizations. OsPDF1A is highly expressed in seedling roots and localizes exclusively to chloroplasts, while OsPDF1B is strongly expressed in mature leaves and dual-localized to both chloroplasts and mitochondria. Notably, OsPDF1B is essential for rice growth and organelle development. Its dual organelle localization, coupled with the lethal chloroplast defects observed in *ospdf1b* knockout mutants, confirms its critical role in chloroplast biogenesis [22].

In the present study, we employed bioinformatics approaches to systematically analyze the *OsPDF* gene family. Specifically, we conducted analyses of phylogenetic relationships, gene collinearity, sequence homology, gene structure, conserved motifs, chromosomal localization, expression patterns, and cis-regulatory elements in promoter regions for all *OsPDF* family members. From this comprehensive gene family investigation, we drew the following conclusions.

### 4.1. Genomic Architecture and Evolutionary Dynamics of OsPDF Genes

During species evolution, the PDF family has conservatively diverged into two subfamilies, PDF1A and PDF1B. OsPDF1A belongs to the former, while OsPDF1B and OsPDF1B2 belong to the latter. Such subfamily divergence reflects the functional diversity of this gene family and is closely associated with their differential subcellular localization as well as organelle-regulatory functions of PDF1B such as chloroplast development.

The three genes of the rice *PDF* family (*OsPDF1A*, *OsPDF1B*, and *OsPDF1B2*) are concentrated on the same chromosome, implying that they coordinately regulate plant growth and development.

Cross-species synteny analysis of *PDF* genes among *Oryza sativa*, *Arabidopsis thaliana*, *Solanum lycopersicum*, *Glycine max*, *Zea mays*, and *Triticum aestivum* revealed that *Oryza sativa* and *Triticum aestivum* (both gramineous monocots) are closely related, with five pairs of orthologous *PDF* gene identified between the two species (Figure 3). This indicates that the function and genomic arrangement of *PDF* genes are conserved during the evolution of gramineous plants. This difference suggests that *PDF* genes have evolved along distinct trajectories after the divergence of monocots and dicots, and such divergence is most likely driven by lineage-specific adaptive requirements for protein maturation in plant organelles (e.g., chloroplasts, mitochondria).

### 4.2. Functional Prediction of OsPDF Gene Family

To explore the potential roles of the *OsPDF* gene family in plant stress responses, we conducted a comprehensive integrative analysis, encompassing cis-regulatory element identification in promoters and expression profiling of *OsPDF* genes. This analysis systematically elucidated the multi-dimensional regulatory mechanisms through which the OsPDF family may mediate plant responses to environmental stresses. Promoter sequence analysis revealed that *OsPDF* genes are enriched with diverse cis-acting elements, including hormone-responsive motifs (e.g., for ABA, MeJA, GA and auxin), growth- and development-related elements (e.g., light-responsive and cell cycle-regulatory motifs), and stress-responsive elements (e.g., those associated with low temperature). These findings collectively suggest that *OsPDF* genes are likely involved in plant response pathways to low temperature, as well as in processes modulated by hormones and growth signals.

To validate these predictive results, we performed qRT-PCR analysis to examine *OsPDF* expression patterns under various stress treatments. The results confirmed that all *OsPDF* genes showed upregulated expression following low-temperature, high-temperature, salt stress, or UV-B treatment (Figure 5). Under salt stress, *OsPDF1A*, *OsPDF1B* and *OsPDF1B2* also exhibited consistently elevated expression relative to the control. Notably, *OsPDF1B* displayed a relatively high expression level compared to the control, which indicates that it may play a more prominent role in the plant’s response to salt stress than the other two *OsPDF* members.

In summary, the integrative analysis and qRT-PCR validation provide evidence for the involvement of the *OsPDF* gene family in multiple stress response pathways. Further investigation into the molecular mechanisms underlying their stress-responsive functions, coupled with functional validation (e.g., via gene knockout or overexpression), will help clarify their precise roles in plant stress resistance. This work also holds potential to provide novel theoretical foundations for molecular breeding strategies aimed at improving stress tolerance in rice.

### 4.3. Limitations and Future Directions

Existing research on plant PDF has primarily focused on the function of the PDF1B subgroup. For instance, in rice (*Oryza sativa*), two independent T-DNA insertion mutants (*pdf1b-1* and *pdf1b-2*) were generated in prior work, with insertions in the third intron and first intron of *OsPDF1B*, respectively. Homozygous *pdf1b*/*pdf1b* plants exhibited distinct phenotypes, including chlorosis (yellowish leaves), severe growth retardation, chloroplast structural damage, and dysregulated expression of organellar genes [14]. In a more recent study on the woody plant *Eucommia ulmoides*, functional analysis of transgenic tobacco overexpressing *EuPDF1B* further confirmed PDF1B’s role. Transgenic tobacco plants overexpressing *EuPDF1B* showed higher plant height, fresh weight, and net photosynthetic rate compared to wild-type plants, accompanied by increased expression of the chloroplast *psbA* gene and denser chloroplast grana thylakoid membranes. This aligns with PDF1B’s conserved function in PSII maintenance—overexpression enhances the synthesis and turnover of the D1 protein, thereby supporting efficient PSII repair and sustaining photosynthetic function [15].

Notably, a key limitation of current PDF research lies in the lack of comprehensive functional characterization of the PDF1A subgroup. In the present study, we observed that *OsPDF1A* (a rice PDF1A member) exhibited altered expression in response to multiple stress treatments, suggesting that it may also play roles in mediating plant stress adaptation. To address this knowledge gap, subsequent research should prioritize functional validation of OsPDF1A by employing CRISPR/Cas9-based genetic manipulation to generate overexpression or knockout mutants, which will be essential to clarify its gene-specific roles in stress responses. Such work will help fill the current gap in our understanding of the functional diversity of the *OsPDF* gene family and provide a more holistic view of PDF-mediated regulatory networks in rice.

## 5. Conclusions

This study systematically characterized the *OsPDF* gene family in *Oryza sativa* through integrated bioinformatics and experimental analyses, shedding light on their genomic features, evolutionary dynamics, and functional roles in abiotic stress responses.

Three *OsPDF* genes (*OsPDF1A*, *OsPDF1B*, *OsPDF1B2*) were identified, clustering into two subfamilies (*PDF1A* and *PDF1B*) and localizing exclusively on rice chromosome 1. Synteny analysis showed closer evolutionary ties between rice and the monocot Triticum aestivum (with five collinear gene pairs) than with dicots, reflecting conserved PDF function in gramineous plants. Motif analysis revealed differences in conserved catalytic motifs (e.g., more Motif3 copies in *OsPDF1A*) that may underpin functional specialization. Biophysical property analysis classified OsPDF1A/1B as alkaline and OsPDF1B2 as acidic, with all three being hydrophilic and moderately thermostable. Promoter cis-element analysis identified hormone-responsive (ABA, MeJA, etc.), growth-related (light, cell cycle), and stress-responsive (low temperature, drought, etc.) elements, suggesting that *OsPDFs* integrate diverse signals for plant development and stress adaptation. The qRT-PCR results confirmed *OsPDFs’* transcriptional responses to abiotic stresses: *OsPDF1A/1B* were upregulated under low temperature; all three genes were activated by heat, salt, and UV-B; and *OsPDF1B* showed significantly strong upregulation under salt stress.

In short, this study clarifies the genomic, evolutionary, and stress-responsive features of the *OsPDF* family in rice. Future work on *OsPDF1A* (a less studied *PDF1A* member) via genetic tools (e.g., CRISPR/Cas9) will help fill the gaps in our understanding of OsPDF functional diversity, supporting rice stress tolerance breeding.

## Figures and Tables

**Table 1:** Information regarding the *OsPDFs*.

Sequence ID	Number of Amino Acid	Molecular Weight	Theoretical pI	Instability Index	Aliphatic Index	Grand Average of Hydropathicity
OsPDF1A	260	27,492.77	8.79	48.69	96.81	−0.043
OsPDF1B	269	29,676.22	8.54	46.86	93.53	−0.142
OsPDF1B2	161	18,006.76	5.47	45.22	97.89	−0.104

## Data Availability

The original contributions presented in this study are included in the article/Supplementary Materials. Further inquiries can be directed to the corresponding author.

## Supplementary Materials

The following supporting information can be downloaded at: https://www.mdpi.com/article/10.3390/cimb48040396/s1.
