# Supplementary material for: Integrated Phylogenomics and Expression Profiling of the Peptide Deformylase Gene Family in Oryza sativa Reveals Their Role in Development and Stress Tolerance
Source: Curr Issues Mol Biol. 2026 Apr 13;48(4):396. doi: 10.3390/cimb48040396 (PMC13115251; doi:10.3390/cimb48040396)
Supplement: Supplementary file 1 [file cimb-48-00396-s001.zip › cimb-4222976-supplementary.pdf]

Supplementary Table S1. List of primers used in this study

Primers for quantitative real-time PCR

**Gene name**	**Sequence (5'-3')**
OsPDF1A	CGCCGTCCTTTCGATCTTCT
	GGGCGTCCATTCCTGTCCAA
OsPDF1B	CGGGTTCGCGTCCTCCTT
	CGCCACCGAGCCCATGC
OsPDF1B2	TGCGGGGAGAGGGAAAAA
	GGGCCGAGGCTGAAGTGAAG
ACTIN	TGTCTTCCCCAGCATTGTCG
	ACGATACCATGCTCGATGGG
